# Bellidifolin Inhibits Proliferation of A549 Cells by Regulating STAT3/COX-2 Expression and Protein Activity

**DOI:** 10.1155/2020/1723791

**Published:** 2020-11-21

**Authors:** Li Yan, Luo Yali, Li Chenghao, Feng Caiqin, Zhu Zhongbo, Ren Weiyu, Ma Yu, Zhou Xiaotian, Wang Biwen, Jin Xiaojie, Liu Yongqi

**Affiliations:** ^1^Provincial-Level Key Laboratory for Molecular Medicine of Major Diseases and the Prevention and Treatment with Traditional Chinese Medicine Research in Gansu Colleges and University, Gansu University of Chinese Medicine, Lanzhou 730000, China; ^2^Key Laboratory of Dun Huang Medical and Transformation, Ministry of Education of the People's Republic of China, Lanzhou 730000, China; ^3^Affiliated Hospital of Gansu University of Chinese Medicine, Lanzhou 730000, China; ^4^College of Basic Medicine, Gansu University of Chinese Medicine, Lanzhou 730000, China; ^5^College of Pharmacy, Gansu University of Chinese Medicine, Lanzhou 730000, China

## Abstract

**Objectives:**

Bellidifolin (BEL) is one type of tetraoxygenated xanthone that is particularly found in *Swertia* and *Gentiana* (Gentianaceae). Despite its broad range of pharmacological activities, it is still unclear whether BEL could be used for lung cancer treatment. Hence, we presently demonstrate the roles of BEL towards the proliferative inhibition of the prototypical A549 lung cancer cells.

**Materials and Methods:**

The antiproliferative activity of BEL was initially verified by cellular experiments. A network pharmacology method was then pursued to assess BEL potential molecular targets from the platform for pharmacological analysis of Traditional Chinese Medicine Systems Pharmacology Database and Analysis Platform (TCMSP). Disease enrichment of potential targets and construction of compound-target-disease network maps were performed based on a total of 20 diseases. Two core targets related to the BEL-mediated effect in A549 cells were obtained by importing potential targets into a protein-protein interaction database (STRING) and also analyzing respective data of related targets into this database. Last, these core targets were examined by in vitro analysis and molecular docking.

**Results:**

CCK8 assays indicated that treatment with 50–100 *μ*m BEL had an inhibitory effect on the proliferation of human A549 lung cancer cells, whereas this effect was time- and concentration-dependent. As control, treatment with 50–100 *μ*m BEL did not inhibit the proliferation of normal lung epithelial cells (BEAS-2b cell line). H&E staining of BEL-treated A549 cells showed that, upon an increase of drug concentration, nuclear condensation and fragmentation were largely observed. Cell cycle analysis showed that in vitro treatment with 75–100 *μ*m BEL could block A549 cells in S and G2 phases. Western blot analyses showed that after 72 hours of BEL treatment, the level of caspase-8/3 in A549 cells increased, and the level of PARP1 decreased in a dose-dependent manner. Network pharmacology analysis also indicated that lung cancer was the major disease susceptible to BEL treatment. At the same time, STAT3 and COX-2 were identified as two core targets of BEL in lung cancer treatment. Functional analyses further revealed that the cytotoxicity effect of BEL in A549 cells potentially involved the STAT3/COX-2 pathway. Moreover, molecular docking analysis indicated that BEL structure properly matches with COX-2 and STAT3 in space shape, thus illustrating the putative molecular mechanism of BEL's anticancer effect.

**Conclusions:**

Based on a series of in vitro analyses, network pharmacology, and molecular docking, the potential mechanism involving the antiproliferative and cytotoxic effects of BEL in lung cancer cells was investigated. Our study may help providing some theoretical basis for the discovery of novel phytotherapy drugs applicable for the treatment of lung cancer.

## 1. Introduction

Lung cancer is one of the most common malignancies with high mortality worldwide [1]. Lung cancer is frequently grouped according to its pathological classification, i.e., nonsmall and small cell lung cancer (NSCLC and SCLC, respectively). NSCLC is considered the most usual type of lung cancer, comprising 85% of all cases [2]. Currently, the main clinical treatment of lung cancer relates to the use of monoclonal antibodies. Nevertheless, this immunotherapy approach frequently leads to side effects, such as allergy, gastrointestinal disorders, and others. Since the large production and purification of monoclonal antibody drugs is also expensive and usually time-demanding, searching for novel therapeutic approaches against this aggressive condition is of seminal importance. In this regard, a number of small molecules and peptides have been characterized as potential inhibitors of lung cancer progression. For instance, anlotinib hydrochloride (AL3818), a novel small molecule that acts as a multitarget tyrosine kinase inhibitor, can inhibit angiogenesis by blocking *CCL2* expression in refractory advanced NSCLC [3]. Still, clinical studies have shown that AL3818 can potentially lead to hypertension-related effects during treatment [4].

A number of studies have recently focused on the potential use of natural components, isolated from traditional Chinese herbal medicine, as novel antitumor drugs [5]. The flavonoid bellidifolin (BEL) is a natural xanthone compound derived from plants of the *Gentiana* species. Previous studies have suggested that BEL may work as an hypoglycemic drug and for the treatment of cardiovascular conditions [6], AIDS [7], and cerebral ischemic injuries [8]. In the context of cancer, particularly NSCLC, no studies have reported the potential use (*f* any) of BEL as a therapeutic agent.

BEL can also drive some distinctive anti-inflammatory effects. For instance, BEL may abrogate inflammatory processes by regulating a number of signaling pathways (i.e., COX-2, NF-*κ*B, MAPKs, and Akt) in RAW 264.7 macrophage cells [9]. Coincidentally, lung cancer-induced inflammation remains the leading cause of mortality among affected patients. At the same time, chronic lung inflammation has been associated with the increased risk to lung cancer [10, 11]. Therefore, the putative anti-inflammatory activity of BEL (among other functions) could be appealing for the treatment of lung cancer.

In the present work, we evaluated the effects and the mechanism of action (MOA) of BEL in NSCLC. In previous cell suppression experiments, we have found that BEL has a significant inhibitory effect in the proliferation of A549 cells (prototypical cell line derived from human lung adenocarcinoma). Hence, here, we combined a variety of biological experiments, network pharmacology technology, and molecular docking methods to validate the putative antitumorigenic activity of BEL in lung cancer and to screen tumor-related protein genes that may integrate BEL-mediated anticancer functions.

## 2. Materials and Methods

### 2.1. Chemical Structural Formula


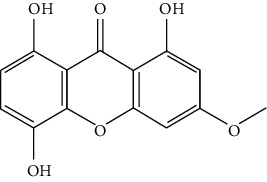


The chemical properties of this compound are as follows:

  CAS number: 2798-25-6,
  Molecular formula: C14H10O6,
  Molecular weight: 274.23,
  Density: 1.586 ± 0.06 g/cm3,
  Melting point: 270°C,
  Boiling point: 580.2 ± 50.0°C,
  Purity: HPLC ≥ 98%.

The method of separation/purification of the drug is not disclosed (drug is commercially available).

#### 2.1.1. Cell Culture and Drug Treatment

Human A549 lung cancer cells were routinely cultured in RPMI 1640 medium (HyClone, Thermo Scientific, MA, USA), while normal human lung epithelial BEAS-2b cells were cultured in F-12K medium (HyClone, Thermo Scientific, MA, USA), both containing 10% fetal bovine serum (catalog # FB15011, CLARK, South America, America) and 1% penicillin/streptomycin. Cells were incubated at 37°C in an atmosphere of 5% CO_2_ and saturated humidity. Cells were eventually passed with 0.25% trypsin (catalog # 25200–056, Gibco). Bellidifolin (BEL; catalog #2798-25-6, purity > 98%, Vicki, Sichuan Province, China) was solubilized in DMSO to make a 10 mm stock solution. Anlotinib (AL3818) (catalog #S8726, Selleck, USA) was also solubilized in DMSO to make a 50 nm stock solution. For wound healing assays, A549 cells were treated with 50, 75, or 100 *μ*m BEL and 2.5 or 5 *μ*m anlotinib. For cell viability analysis, both BEAS-2b and A549 cells were, respectively, treated with 25, 50, 75, or 100 *μ*m BEL, while A549 cells were alternatively treated with 2.5, 5, 10, or 20 *μ*m anlotinib. For hematoxylin and eosin (H&E) staining, A549 cells were treated with 50, 75, or 100 *μ*m BEL and 2.5 or 5 *μ*m anlotinib. For the remaining tests, A549 cells were treated with BEL at concentrations of 50, 75, or 100 *μ*m.

#### 2.1.2. Cell Proliferation Analysis

CCK-8 viability assay (Dojindo Molecular Technologies, China) was used to assess BEL antitumor activity. For this, A549 cells were resuspended with 0.25% trypsin and inoculated into 96-well plates at a density of 3 × 10^3^ cells per well. The final BEL concentrations used per condition were 25, 50, 75, or 100 *μ*m. BEAS-2b cells were treated with BEL at concentrations of 25, 50, 75, or 100 *μ*m. The effect of BEL on the survival rate of normal human lung epithelial BEAS-2b cells was reevaluated using the CCK-8 assay. Amount of viable cells was estimated according to absorbance (Abs) at 450 nm using a microplate reader (IMark, Bio-Rad). The cell inhibition rate was calculated as the following: inhibition rate (%) = ((Abs control group − Abs experiment group) / (Abs control group − Abs blank group)) × 100%. Cell survival was quantified as follows: survival (%) = ((Abs experimental group − Abs blank group) / (Abs control group − Abs blank group)) × 100%. All experiments were performed in triplicates.

#### 2.1.3. Wound Healing Assay

Cells were plated into a 6-well dish at 5 × 10^5^ cells per well. After cell attachment, a 1 mL gun head was used to scratch a horizontal line in the center of each well. Cells were further treated with 0 (blank), 50, 75, or 100 *μ*m BEL, and 2.5 or 5 *μ*m anlotinib (total of six groups). Within 72 hrs after the scrape line was made, wound healing was observed, and representative scrape lines were imaged using an optical microscope (IX81, Olympus, Japan). ImageJ (NIH) was utilized to calculate and measure the area between the sides of the scratch.

#### 2.1.4. H&E Staining

Control and treated cells were plated into coated-glass slides and cultured for 3 days. Afterward, cell media was removed, and cells were washed twice with 1x PBS. Thereafter, cells were fixed with cold 70% ethanol for 20 mins and then washed with 1xPBS twice again. Hematoxylin staining was performed for 15 mins, followed by 3 washes with 1xPBS. Subsequently, eosin staining was performed for another 15 mins, followed by a new round of 3 washes with 1xPBS. Stained cells were dried at room temperature and then mounted with neutral gum. Cell morphology was examined under an optical microscope.

#### 2.1.5. Apoptosis and Cell Cycle Analyses

Cell apoptosis was evaluated according to manufacturer's protocol (BD Biosciences Pharmingen, UK). For this, cells of each treatment group were washed twice with precooled PBS on ice. After centrifugation, cells were resuspended in 1x binding buffer and stained with Annexin V/PI for 15 mins at room temperature. Flow cytometry analysis was performed within 1 hour after staining.

Cell cycle analysis was performed according to manufacturer's instructions (Multi Sciences, China). In brief, cells were incubated for 3 days and resuspended thereafter. Cell number was counted, and respective cell suspension was adjusted to 1 × l0^6^ cells/ml. Cells were further centrifuged, and the supernatant was removed. Cell pellets were washed once with 1xPBS, and after centrifugation, the supernatant was discarded. One milliliter of Reagent A (DNA staining solution) and 10 dL of Reagent B (permeabilization solution) was added per sample. Respective samples were then vortexed/mixed for 5–10 s before ﬂow cytometry was proceeded.

#### 2.1.6. Western Blotting

After cell lysis, protein concentration was determined using a BCA protein quantitative kit. Protein lysates were resolved by SDS-polyacrylamide gel electrophoresis and then transferred to a PVDF membrane. Thereafter, membrane was blocked with 5% *w*/*v* nonfat dry milk in 1xTBST for 2 hrs and then incubated with respective primary antibodies (1 : 1,500 dilution) overnight at 4°C. Primary antibodies against the following proteins were used: PARP1, caspase-3/8 (Abcam, Cambridge, UK), STAT3/P-STAT3 (Abcam, Cambridge, UK), COX-2 (Abcam, Cambridge, UK), and GAPDH (Abcam, Cambridge, UK). Afterward, the membrane was washed 2-3 times with 1xTBST and then incubated with the respective secondary antibody (goat anti-rabbit horseradish peroxidase (HRP) conjugated antibody, 1 : 5,000 dilution) (Abcam, Cambridge, UK) for 2 hrs at room temperature. After addition of HRP substrate, membranes were examined using an Image acquisition and analysis system (ChemiDoc-610, UVP, UK). The band signal of each target protein was quantified by ImageJ and normalized according to respective GAPDH levels.

#### 2.1.7. Real-Time qPCR

Total RNA was extracted according to reagent protocol (TRIzol™ reagent, Thermo, USA). First-strand cDNA synthesis was performed using PrimeScript® RT master mix. Quantitative RT-PCR was carried out using the respective kit (Yesheng Biotechnology, Shanghai), according to the manufacturer's instructions, using GAPDH as a control. RNA expression levels were assessed using the 2^−ΔΔCT^ method. Each experiment was independently executed at least three times. The primer sequences and the content of respective qPCR reactions are listed (Tables 1 and 2).

### 2.2. Network Pharmacology

#### 2.2.1. BEL Target Prediction

TCMSP and Swiss Target Prediction databases (http://swisstargetprediction.ch/) were utilized to retrieve BEL-related targets. For this, UniProt database was also accessed by unifying all identified targets using respective UniProt IDs (thus deleting duplicated items) to ultimately establish a target database.

#### 2.2.2. Disease Prediction

Potential BEL-related targets were entered into the DAVID database (https://david.ncifcrf.gov/home.jsp) for disease enrichment collation of these respective molecules. Thereafter, a compound-target-disease network was designed using Cytoscape 3.6.1 according to the first twenty diseases correlated with BEL-related targets.

#### 2.2.3. Construction and Analysis of PPI Network

The presumptive BEL-related target database was imported into the String database. To construct a protein-protein interaction map based on these targets, “*Homo sapiens*” was selected as the sole analyzed species. Resulting analytical data were further imported into the Cytoscape 3.6.1, using a network analyzer of modules for analytical (degree) and indirect (betweenness centrality) centralities. The range of both values was set to be greater than the average value, resulting in the definition of BEL core targets.

### 2.3. Molecular Docking

#### 2.3.1. Receptor Preparation

Molecular docking was achieved using the Schrodinger 9.4.5 software. The STAT3 protein structure was obtained from respective compounds (PDB code: 6QHD [12], resolution ratio 2.85 Å), while the COX-2 structure was acquired from the crystal complex of cyclooxygenase-2 (prostaglandin synthetase-2) and the selective inhibitor SC-558 (PDB code: 6COX [13] at resolution ratio of 2.80 Å). Protein structure was hydrogenated using protein preparation wizard plates, protonated at pH = 7, and finally optimized under the OPLS-2005 force field.

#### 2.3.2. Ligand Preparation

Four small ligand molecules were utilized in the docking process, namely, bellidifolin (BEL), STAT3 inhibitor BP-1-102 [14], anlotinib, and COX-2 inhibitor celecoxib [15]. Structural optimization was performed using LigPrep plates. The ionization state of each ligand three-dimensional (3D) structure and tautomerization was generated using the Epik module and retention of its initial chiral. All other parameters were applied as default.

#### 2.3.3. Determination of Binding Site

Combined STAT3 pockets were searched in the literature and then confirmed using receptor grid generation plates. COX-2 bags were identified via Schrodinger's SiteMap module.

#### 2.3.4. Docking Score

All small molecule ligands were docked with their corresponding receptors using an extra precision (XP) scoring model. Docking results were scored accordingly.

## 3. Statistical Treatment

The SPSS 18.0 statistical software was used for statistical analysis. Measured data were expressed as mean ± SD. One-way ANOVA was performed if the data presented a normal distribution. In the absence of a normal distribution, multiple independent samples from a nonparametric test were assessed by the Kruskal–Wallis *H* test. A *P* value lower than 0.05 was set as a cutoff of statistical significance.

## 4. Results

### 4.1. Effect of BEL Treatment on the Proliferation of Human A549 Lung Cancer Cells

**Figure 1:**
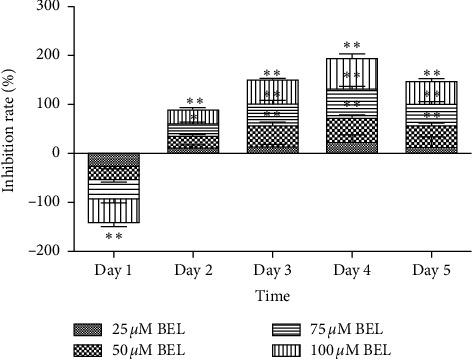
Columnar section of the percentage (rate) of growth inhibition for lung cancer cells treated with different concentrations of BEL. Human A549 lung cancer cells were treated with 25, 50, 75, or 100 *μ*m BEL for 5 days (*n* = 3). Data are presented as mean ± SD. Results were normalized according to the inhibition of cells treated with 25 *μ*m BEL. ^*∗*^*P* < 0.05; ^*∗∗*^*P* < 0.01.

To evaluate the effect of BEL treatment on the growth/proliferation of lung cancer cells in vitro, A549 cells were treated with increasing doses of BEL concentration at different times. The inhibitory effect of BEL towards the growth of A549 cells increased significantly over time. At 72 hours of BEL treatment, growth inhibition was more evident at 50–100 *μ*m BEL. The one-way ANOVA test showed a significant difference when comparing these results with those obtained from cells treated with 25 *μ*m BEL (*P* < 0.01). Thus, a 72 hour timepoint was selected to examine the inhibitory effect of BEL in lung cancer cells (Figure 1). These data indicate that BEL has a potent antiproliferative activity in human lung cancer cells.

### 4.2. Effect of BEL Treatment on the Proliferation of Normal Human Lung Epithelial BEAS-2b Cells

**Figure 2:**
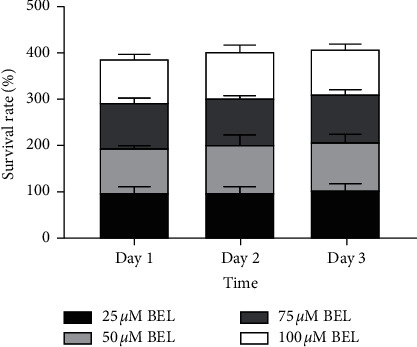
Columnar section of survival rates for lung epithelial BEAS-2b cells treated with BEL at different concentration. As indicated, BEAS-2b cells were stimulated with 25, 50, 75, or 100 *μ*m BEL for 3 days (*n* = 3). Data are presented as mean ± SD. Results were normalized according to the survival of cells treated with 25 *μ*m BEL. ^*∗*^*P* < 0.05; ^*∗∗*^*P* < 0.01.

To evaluate whether similar doses of BEL (50–100 *μ*m concentration) could also impact the growth/proliferation of normal lung cells in vitro, human lung epithelial BEAS-2b cells were treated with same BEL concentrations No cytotoxic or antiproliferative effect was noticed when BEAS-2b cells were treated with 25–100 *μ*m BEL for 72 hrs (*P* > 0.05) (Figure 2), thus indicating that this dose range could be specifically used against lung cancer cells.

### 4.3. Wound Healing Analysis of BEL-Treated A549 Cells

**Figure 3:**
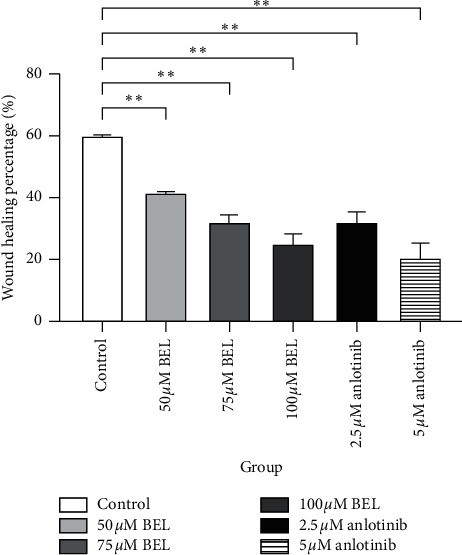
Columnar section illustrating the percentage of wound healing in vitro for nontreated (control) or drug-treated lung cancer cells. A549 cells were treated with 50, 75, or 100 *μ*m BEL or 2.5 or 5 *μ*m anlotinib (positive control) for 3 days (*n* = 3). Data are presented as mean ± SD. Percentages were obtained upon comparison with the control group. ^*∗*^*P* < 0.05; ^*∗∗*^*P* < 0.01.

**Figure 4:**
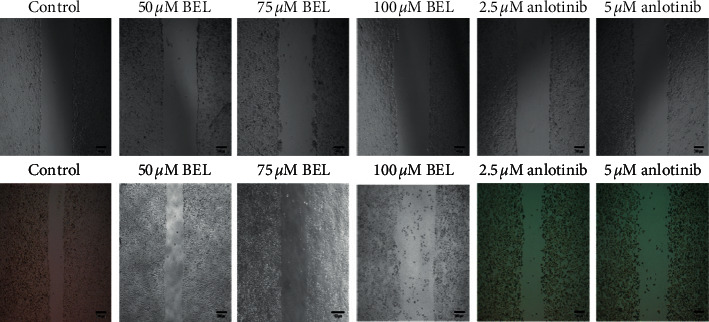
Imaging of the scratch test for respective lung cancer cell groups. A549 cells were treated with 50, 75, or 100 *μ*m BEL or 2.5 or 5 *μ*m anlotinib for 3 days (*n* = 3). Top images are related to day 0, while those at the bottom are from 3-day treatment. Nontreated cells were used as control. Scale bars = 200 *μ*m.

To verify whether BEL could affect the migration capacity of lung cancer cells in vitro, a scratch test (wound healing assay) was performed with cultured A549 cells treated with BEL. After 3 days of drug treatment, cell migration over the scratched plate surface was significantly inhibited when compared to nontreated (control) cells (*P* < 0.01). Particularly, cell treatment with 100 *μ*m BEL and 5 *μ*m anlotinib (positive control) showed the most significant reduction on wound healing in vitro (Figures 3 and 4).

### 4.4. Morphological Analysis (H&E Staining) of BEL-Treated A549 Cells

**Figure 5:**
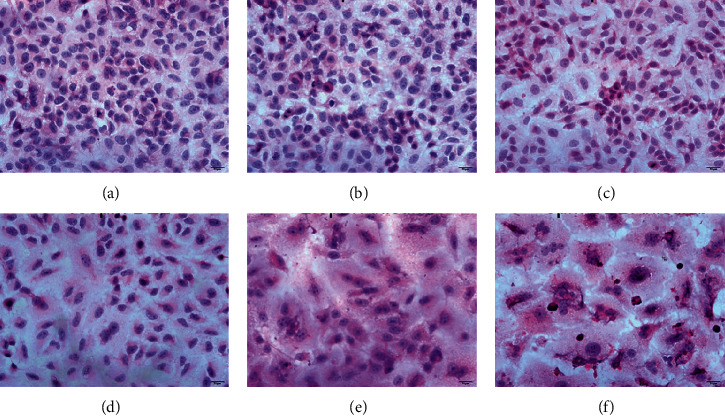
Morphological changes of BEL-treated lung cancer cells after 3 days of induction. Cell morphology was examined by H&E staining. For this, A549 cells were treated with 50 *μ*m, 75 *μ*m, or 100 *μ*m BEL. Nontreated and anlotinib-treated cells (2.5 or 5 *μ*m concentration) were included as negative and positive controls, respectively (*n* = 3). Scale bars = 20 *μ*m, (a) control, (b) 50 *μ*m BEL, (c) 75 *μ*m BEL, (d) 100 *μ*m BEL, (e) 2.5 *μ*m anlotinib, and (f) 5 *μ*m BEL anlotinib.

In order to confirm that BEL possesses some antitumor activity against lung cancer in vitro, H&E staining was performed to evaluate any morphological changes of A549 cells after treatment with BEL for 3 days. The impact of these changes was evaluated according to those observed in nontreated and anlotinib-treated cells (negative and positive control, respectively). Nontreated cancer cells (control) presented more pathological karyokinesis, while the drug-treated cells showed more prominent nuclear concentration, deep staining, increased apoptosis, and necrosis. In addition, cell mass increased proportionally to the drug concentration (BEL and anlotinib (positive control)), with more significant nuclear concentration and death-related events (Figure 5).

### 4.5. Apoptosis Analysis of BEL-Treated A549 Cells Using Flow Cytometry

**Figure 6:**
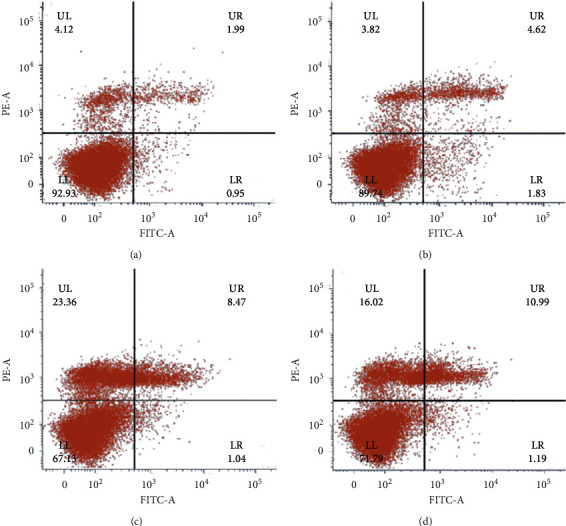
Changes on the apoptosis rate of BEL-treated lung cancer cells. A549 cells were treated with 50, 75, or 100 *μ*m BEL concentration for 3 days (*n* = 3). Nontreated cells were also analyzed as a control group: (a) control, (b) 50 *μ*m BEL, (c) 75 *μ*m BEL, and (d) 100 *μ*m BEL.

**Figure 7:**
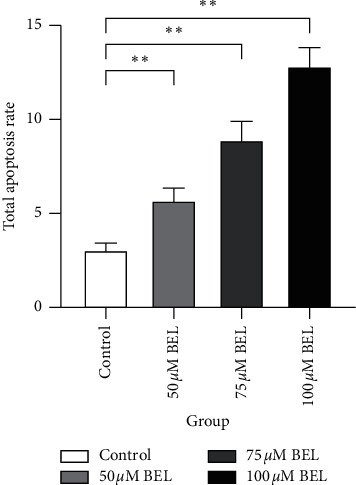
Columnar section illustrating the changes on total apoptosis of BEL-treated lung cancer cells. A549 cells were treated with 50, 75, or 100 *μ*m BEL for 3 days (*n* = 3). Data are presented as means ± SD, upon comparison with nontreated (control) cells. ^*∗*^*P* < 0.05; ^*∗∗*^*P* < 0.01.

In order to verify whether BEL-mediated inhibition of A549 cell proliferation was due to apoptosis induction, we evaluated the apoptosis rate of A549 cells after three days of BEL treatment. After treatment, cells were processed and stained with Annexin V/PI for further flow cytometry. Results indicated that the increased apoptosis rate of BEL-treated A549 cells was proportional to the drug concentration, reiterating the pattern of a dose-dependent effect. Upon reaching a concentration of 100 *μ*m BEL, the apoptosis rate was significantly distinguishable (Figures 6 and 7).

### 4.6. Cell Cycle Analysis of BEL-Treated A549 Cells Using Flow Cytometry

**Figure 8:**
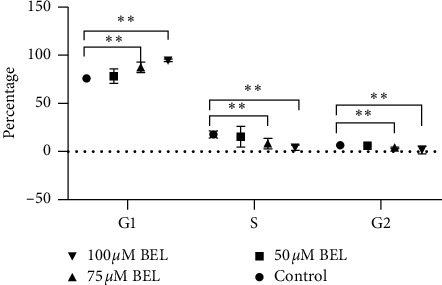
Cell cycle profile of BEL-treated lung cancer cells. A549 cells were treated with 50, 75, or 100 *μ*m BEL for 3 days (*n* = 3). Data are presented as mean ± SD, upon comparison with nontreated (control) cells. ^*∗*^*P* < 0.05; ^*∗∗*^*P* < 0.01.

Cell proliferation is known to be closely related to cell cycle progression. Our results indicated that the proportion of BEL-treated A549 cells (50 *μ*m concentration) at G1, S, and G2 phases was not significantly different from the one observed with nontreated (control) A549 cells (*P* > 0.05). Still, a statistical difference was noticed when comparing the control cells and those treated with 75 *μ*m BEL. In this case, the proportion of cells in the G1 phase increased, while the rates of cells in the S phase decreased after BEL treatment (*P* < 0.01). A significant distinction between control cells and those treated with 100 *μ*m BEL was also observed. However, a more prominent trend of cells at G1 and S phases and a decrease on the rate of cells at the G2 phase were observed upon treatment with 100 *μ*m BEL (*P* < 0.05) (Figure 8).

### 4.7. Caspase-8/Caspase-3/RAPA1 Protein Levels in BEL-Treated A549 Cells

**Figure 9:**
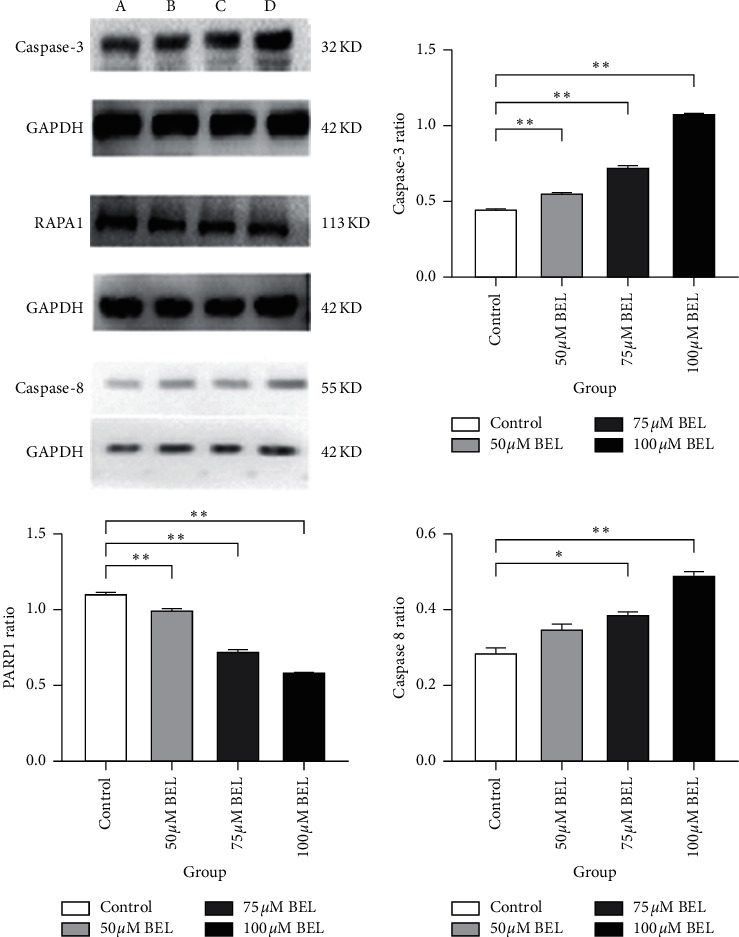
Caspase-8, caspase-3, and PARP1 protein levels in BEL-treated lung cancer cells. A549 cells were treated with 50 *μ*m (B), 75 *μ*m (C), or 100 *μ*m (D) BEL for 3 days (*n* = 3). Western blot analysis was performed using respective antibodies (as indicated). Nontreated cells were also evaluated as control (A). Signal intensity of caspase-8, caspase-3, and PARP1 blots was quantified, and respective protein ratio (versus control cells) was plotted accordingly. Data are presented as mean ± SD. ^*∗*^*P* < 0.05; ^*∗∗*^*P* < 0.01.

In order to further verify whether BEL can induce apoptosis of A549 cells, three major apoptosis response regulators (namely, caspase-8, caspase-3, and PARP1) were analyzed by Western blotting. As shown in Figure 9, upon treating A549 cells with increasing doses of BEL, the level of caspase-8/3 increased, while PARP1 levels decreased, in a dose-dependent manner (Figure 9).

### 4.8. Network-Based Pharmacological Analysis

#### 4.8.1. Prediction of BEL-Related Targets

Based on a network pharmacology approach, we further explored BEL-related targets that could be relevant for treating diseases. For this, we initially constructed a network diagram of BEL's mechanism of action (MOA), aiming to uncover targets that may support the antiproliferative activity of BEL in lung cancer cells. Upon Swiss Target Prediction and TCMSP database retrieval and screening, a total of 24 protein targets were obtained (after removing duplicates). From this list, 11 potential targets were validated by String database screening. The topological parameter analysis of these BEL-related targets is shown in Table 3.

#### 4.8.2. Disease Prediction

**Figure 10:**
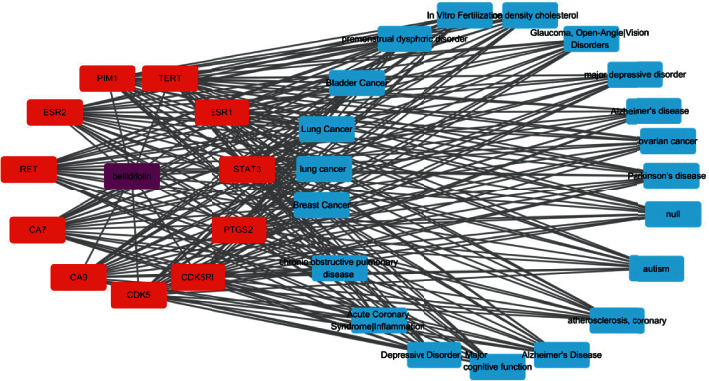
Compound-target-disease network. Purple box indicates BEL, while red boxes indicate potential targets involved in BEL. Light blue boxes correspond to major diseases impacted by BEL-related targets.

As indicated in Table 4, the most prominent diseases (total of 5) linked to potential BEL-related targets were sorted according to their *P* values (the smaller the *P* value, the stronger the significance). Consistently, lung cancer was identified as the top enriched disease (Table 4). According to this data, a compound-target-disease network was further constructed (Figure 10).

#### 4.8.3. Construction and Analysis of PPI Network

**Figure 11:**
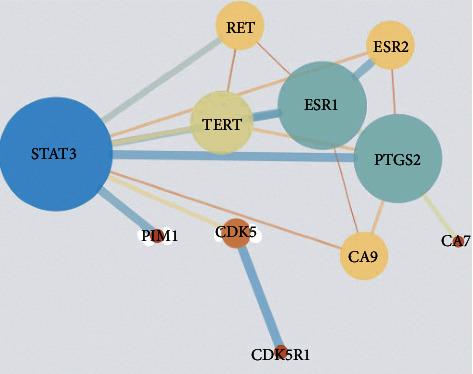
Protein-protein interaction map of potential BEL-related targets. The ball shape symbols (in different colors) represent putative targets. The straight lines indicate the respective connection between targets.

Previous network data were imported into the Cytoscape, using degree and betweenness centrality values above the average as screening criteria. The target interaction diagram (Figure 11) contained a total of 11 circular nodes representing all predicted targets and 16 sided lines representing the association between these targets. As indicated in Figure 11, two core targets were obtained, prostaglandin endoperoxide synthase 2 (PTGS2/COX-2) and STAT3 (degree value was proportional to the size and color of the node area).

We have shown that BEL can effectively inhibit the proliferation and migration of lung cancer cells and promote lung cancer cell apoptosis. According to network pharmacology, the core target STAT3/COX-2 of BEL has been identified. Therefore, the inhibitory effect of BEL on STAT3/COX-2 was further evaluated. In essence, research on STAT3/COX-2 has been regarded as an effective method for treating lung cancer. Therefore, we further verified by WB, qPCR, and other techniques whether BEL may inhibit tumor cell growth through the STAT3/COX-2 pathway.

#### 4.8.4. STAT3 and COX-2 Levels in BEL-Treated A549 Cells

STAT3 is a STAT family protein, which is involved in the occurrence of tumors. Specifically, STAT3 plays an important role in the progression and metastasis of SCLC [16]. STAT3 activates tyrosine or serine kinase to phosphorylate STAT3 (p-STAT3). Uncontrolled STAT3 phosphorylation can lead to uncontrolled cell proliferation and malignant transformation, and thus promote the occurrence of lung cancer [17]. Increased expression of cyclooxygenase 2 occurs frequently in human lung cancers, specifically in adenocarcinomas [18].

**Figure 12:**
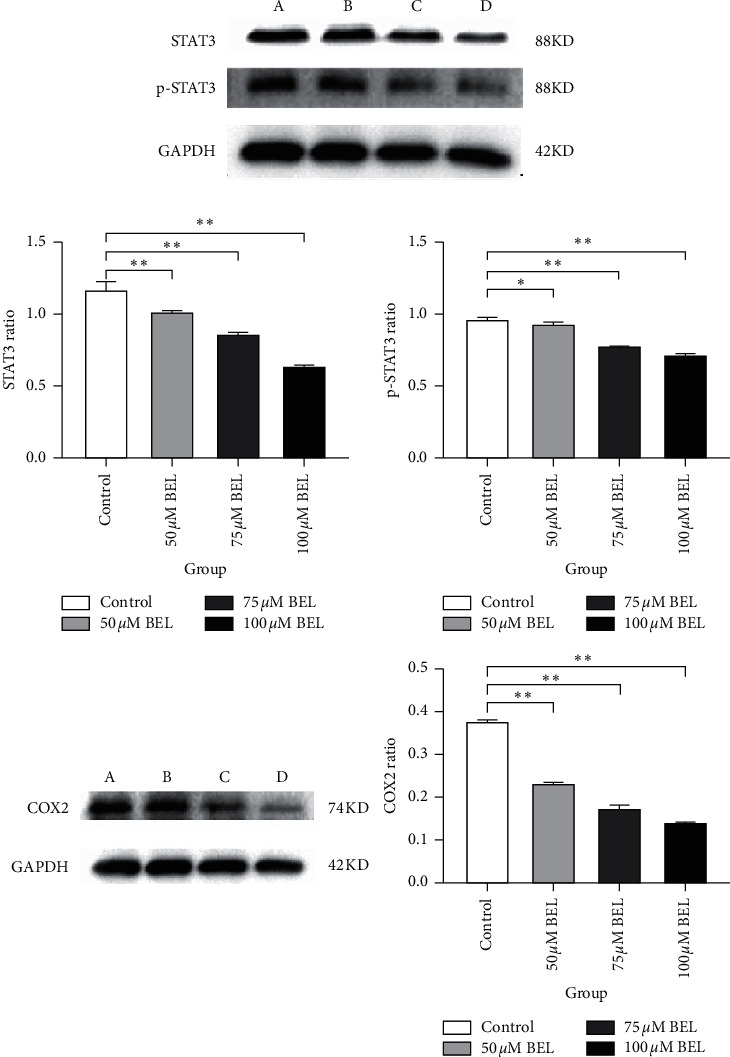
STAT3/p-STAT3 and COX-2 protein levels in BEL-treated lung cancer cells. A549 cells were treated with 50 *μ*m (B), 75 *μ*m (C), and 100 *μ*m (D) BEL for 3 days (*n* = 3). Western blot analysis was performed using respective antibodies (as indicated). Nontreated cells were also evaluated as control (A). Signal intensity of STAT3/p-STAT3 and COX-2 bands was quantified, and respective protein ratio (versus control cells) was further plotted. Data are presented as mean ± SD. ^*∗*^*P* < 0.05; ^*∗∗*^*P* < 0.01.

In order to verify the role (s) of STAT3 and COX-2 towards the antiproliferative activity of BEL in lung cancer cells, Western blot analyses were performed to examine STAT3/p-STAT3 and COX-2 levels in A549 cells after 3 days of BEL treatment. As indicated in Figure 12, STAT3/p-STAT3 and COX-2 levels gradually reduced in response to increasing BEL concentrations in vitro (*P* < 0.01).

#### 4.8.5. Expression Profile of STAT3/COX-2 Signaling Partners in BEL-Treated A549 Cells

**Figure 13:**
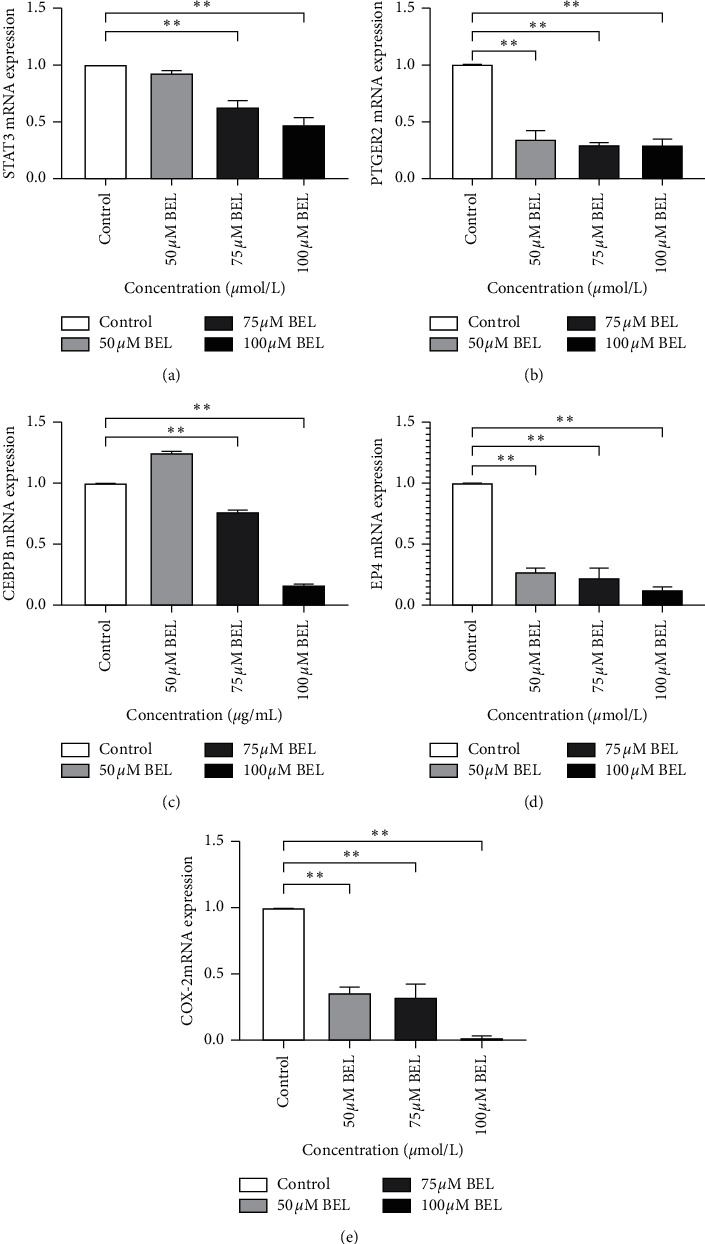
Expression analysis of STAT3/COX-2 partners in BEL-treated lung cancer cells. Graphs A, B, C, D, and E illustrate the relative expression of STAT3, PTGER2, EP4, COX-2, and CEBPB, respectively, in A549 cells treated (or not) with different amounts of BEL (50, 75, or 100 *μ*m) for 3 days (*n* = 3). Expression levels in nontreated cells were assessed as control. Data are presented as mean ± SD, after comparison to the expression in control cells (mean value = 1). ^*∗*^*P* < 0.05; ^*∗∗*^*P* < 0.01.

As shown in Figure 13, the expression of STAT3, COX-2, and some signaling partners (i.e., PTGER2, EP4, and CEBPB) diminished in A549 cells after treatment with increasing concentrations of BEL. Nevertheless, changes on gene expression upon 50 *μ*M BEL treatment were not statistically significant (*P* > 0.05) (Figure 13).

### 4.9. Molecular Docking

Due to their key roles in cancer development and progression, COX-2 and STAT3 have become attractive targets for the development of novel cancer therapeutics. Particularly, the development of inhibitors blocking STAT3 transcriptional activity appears to be a promising therapeutic approach against cancer [19–22]. In order to test whether BEL could directly block the expression of STAT3 and/or COX-2 to ultimately achieve an anticancer role, we pursued some molecular docking analyses to evaluate a putative interaction among these molecules and/or related drug derivatives.

BP-1-102 is a small-molecule STAT3 inhibitor that has a great potential for clinical development [23]. Other compounds already described on the literature, such as SH5-07, SH4-54 [24], and S3I-1757 [25], are derivatives obtained by modification of the original BP-1-102 scaffold structure. Here, we selected BP-1-102 as a positive control for the docking analysis of BEL and STAT3 molecules.

Bextra, celecoxib, and rofecoxib are three COXTHI-2 inhibitors that have been approved by the Food and Drug Administration (FDA, USA) for clinical use, but unfortunately, Bextra and Vioxx have been subsequently withdrawn from the market due to safety and side effect issues [26]. Therefore, here, we also included celecoxib (a selective nonsteroidal anti-inflammatory drug) as a positive control in the molecular docking of BEL and COX-2.

#### 4.9.1. Interaction Analyses of BEL with STAT3 and COX-2 Structure

The SH2 domain of the STAT3 protein is a main target of related small molecule inhibitors. Hence, three pocket-type subdomain structures of STAT3 [27] were selected as binding pockets, which included the pTys705 binding site (Lys591, Arg609, Ser611, and Ser613), Leu706, and the side pocket (Il597, Leu607, Thr622, and Ile634).

**Figure 14:**
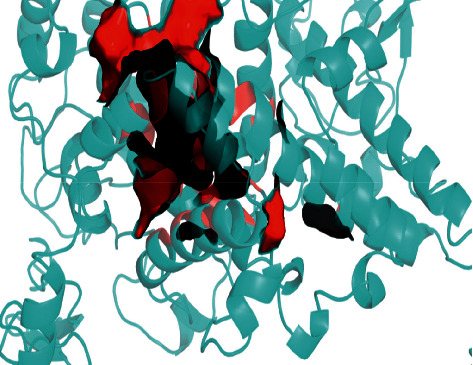
6COX binding pocket. The interacting protein region is indicated in indigo, while the sitemap-14-site-3 site is shown in red.

According to Schrodinger's SiteMap module, COX-2 protein structure (PDB ID: 6COX) was simulated by protein-protein binding interfaces. By default, shallow binding sites were selected, and the scoring of protein-protein binding interface was obtained (Table 5). It was observed that the score of sitemap-14-site-3 was the highest one (1.258) and, therefore, determined as an active pocket. The generation of lattice points remained the default setting. The location of the sitemap-14-site-3 receptor 6 COX was assessed by PyMOL software (Figure 14).

Sitemap-14-site-3 contained a series of residues, including Gln192, Leu93, Thr94, Ser530, Leu531, Phe205, Leu352, Ser353, Leu534, Gly354, Tyr355, Arg513, Pro514, Leu359, Ala516, Ile517, Phe518, Phe381, Leu384, Tyr385, Trp387, Met113, Met522, Val116, Val523, Val344, Gly526, Ala527, Tyr348, Val349, Hie90, and Arg120. Among these positions, Ser530, Tyr355, Tyr385, Tyr348, and Arg120 have been consistently reported in the current literature [28, 29].

#### 4.9.2. Docking Analysis of BEL and STAT3/COX-2

**Figure 15:**
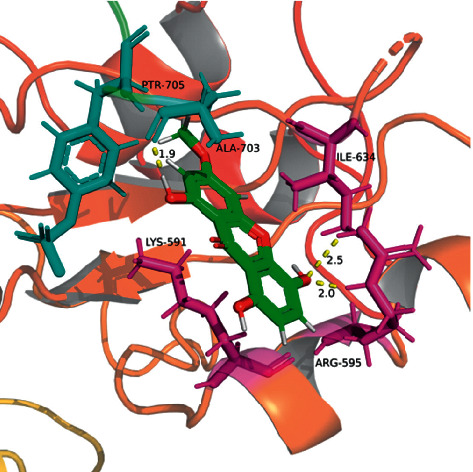
3D diagram of interaction between BEL and STAT3 (PBD: 6QHD). BEL structure is shown in green. The amino acid residues associated with the A-chain binding pocket of 6QHD are indicated in purple. The amino acid residues associated with the B-chain binding pocket of 6QHD are indicated in indigo. Yellow dashed lines indicate the distance of the hydrogen bonds.

**Figure 16:**
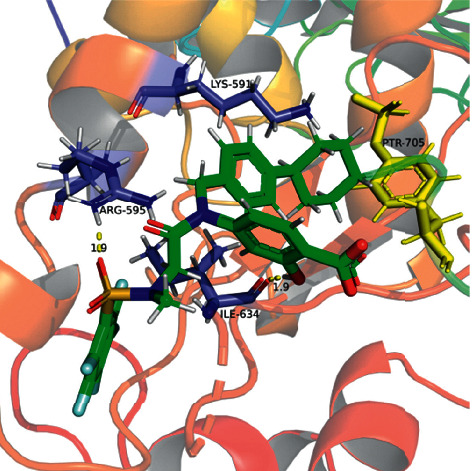
3D diagram of interaction between BP-1-102 and STAT3. BP-1-102 structure is shown in green. The amino acid residues associated with the A-chain binding pocket of 6QHD are indicated in blue. The amino acid residues associated with the B-chain binding pocket of 6QHD are indicated in yellow. Yellow dashed lines indicate the distance of the hydrogen bonds.

**Figure 17:**
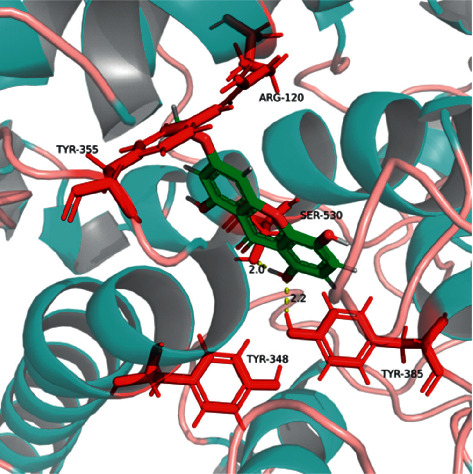
3D diagram of interaction between BEL and COX-2 (PBD: 6COX). The structures of 6COX and BEL are, respectively, shown in indigo and green. The amino acid residues associated with the binding pocket are indicated in red, whereas the yellow dashed lines illustrate the distance of the hydrogen bonds.

**Figure 18:**
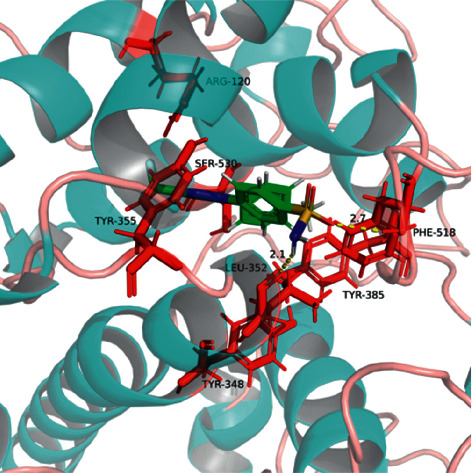
3D diagram of interaction between celecoxib and COX-2. The structures of 6COX and celecoxib are, respectively, shown in indigo and green. The amino acid residues associated with the binding pocket are indicated in red, whereas the yellow dashed lines illustrate the distance of the hydrogen bonds.

On the basis of XP docking, we compared the scores of the corresponding positive control small molecules, related to each receptor, and BEL. As a result, we found that BEL had the possibility of docking with both STAT3 and COX-2 (Tables 6 and 7, Figures 15–18). According to the molecular interaction mode between BEL, BP-1-102, and STAT3, BEL formed hydrogen bonds with Ala703 and Arg595. Moreover, BEL and A-chain Lys591/Arg595 produced a *π*-*π* effect. BEL and Ile634 produced hydrophobic effects. BEL and phosphorylated Tyr705 (Ptr705) produced static electricity. BP-1-102 hydrogen bond was established with Arg595/Ile634. BP-1-102 was also able to generate static electricity in the presence of Lys591 and Ptr705.

According to the molecular interaction mode between BEL, celecoxib, and COX-2, BEL formed hydrogen bonds with Tyr385 and Ser530. BP-1-102 and Arg120 produced a *π*-*π* effect. BEL and Tyr348/Tyr355 produced hydrophobic effects. Celecoxib formed hydrogen bonds with Phe518 and Leu352. Celecoxib and Arg120 produced a *π*-*π* effect, while celecoxib and Ser530 produced some polar effect. Celecoxib and Tyr355/Tyr385/Tyr348 produced hydrophobic effects.

## 5. Discussion

Chronic inflammation is usually considered a prerequisite for a series of cancers [30]. In fact, anti-inflammatory therapies have shown efficacy in cancer prevention and treatment [31]. BEL is a major biologically active ingredient from *Swertia* L and *Gentianella acuta* (*Gentianella*), which has a variety of pharmacological activities. Previous studies have shown that BEL can specifically inhibit COX-2 in an inflammatory response model (RAW 264.7 macrophages treated with LPS) [9]. In the present study, we have observed that BEL can induce S/G2 phase arrest and activate caspase-8/3 in vitro, thus significantly inhibiting the proliferation and cell migration of human A549 lung adenocarcinoma cells. Using network pharmacology and protein docking experiments, we further verified that BEL may play an anticancer effect by directly interfering STAT3 and COX-2 pathways.

A variety of inflammatory mediators, such as STAT3 and COX-2, are involved in the pathogenesis of inflammatory diseases [32]. The release of a large number of inflammatory mediators can promote tumor progression and migration [33]. Therefore, inhibiting the production of these proinflammatory mediators may be an effective tool to counterattack tumor disease development and progression. STAT3 has been closely related to lung cancer. In fact, transduction of the STAT3 signaling pathway appears to significantly alter the biology of lung cancer cells [34]. High *STAT3* expression may serve as a prognostic indicator or therapeutic target for the NSCLC patients [35, 36]. Lu and colleagues have found that HJC0152, an O-alkylamino-tethered niclosamide derivative, can affect NSCLC by inhibiting STAT3 both in vitro and in vivo [37]. Sun and colleagues have also discovered that 2- hydroxy-3-methylanthraquinone can inhibit lung cancer cell growth and invasion by downregulating IL-6-induced JAK2/STAT3 pathways [38]. As one of the downstream effects of STAT3, increased COX-2 expression has been correlated with the occurrence, development, invasion, and acquisition of a metastatic phenotype in lung cancer [39]. Therefore, COX-2 may also be considered as a target for NSCLC therapy [40]. Han and colleagues have demonstrated that tumor associated macrophages (TAMs) have the ability to promote the migration and invasion of osteosarcoma (OS) cells by activating the STAT3/COX-2 axis and inducing epithelial mesenchymal transition (EMT) [41]. Zhang and colleagues have shown that celecoxib can enhance the sensitivity of NSCLC cells to radiation-induced apoptosis by downregulating COX-2 signaling [42]. In the present study, we discovered that BEL can also inhibit lung cancer by inhibiting the expression of COX-2 and STAT3.

COX-2 has the ability to catalyze the conversion of arachidonic acid to prostaglandin h2 (PGH2), which is then converted to prostaglandin E2 (PGE2) by prostaglandin E (PGE) synthase. PGE2 transmits downstream signals by binding to *G* protein-coupled receptor of transmembrane domain EP4. Activation of PGE2 by EP2/EP4 cascades may have a positive role in regulating PD-1 levels of infiltrating CD8^+^ T cells, leading to immune tolerance in the lung cancer microenvironment [43]. STAT3 can also indirectly activate COX-2 by activating the transcription factor C/EBP-*β*. Studies have demonstrated that C/EBP-*β* has an important function in the tumorigenesis and development of a number of malignancies, such as prostate [44], gastric [45], and skin [46] cancers. In this regard, saikosaponin D, an active triterpene saponin, appears to inhibit COX-2 expression by downregulating phospho-STAT3 and C/EBP-*β*. These results suggest that this STAT3/C/EBP-*β*/COX-2 pathway axis and downstream-related factors (i.e., PGE2 and EP4) are closely related to lung cancer and other types of tumors.

**Figure 19:**
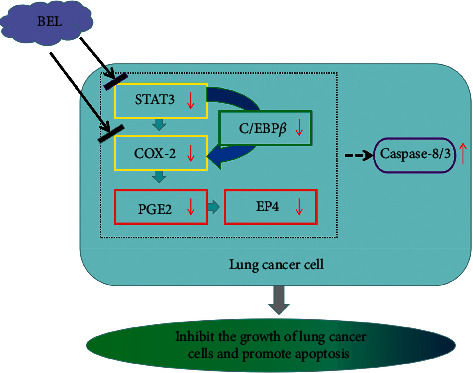
Hypothetical mechanism of BEL-mediated antiproliferative activity in lung cancer cells. T-shaped arrows indicate inhibition, dotted line indicates indirect effect, and solid red arrows indicate downregulation.

Activation of these aforementioned pathways may also lead to the occurrence/promotion of apoptosis. In the apoptotic pathway, caspase-8 regulates the initiation and amplification of apoptosis, while caspase-3 is involved in the execution of apoptotic effects and cell death. Indeed, caspase-3 is considered the most important terminal cleaving enzyme in the process of apoptosis. PARP shearing is considered an important indicator of cell apoptosis and frequently serves as an indicator of caspase-3 activation. Ramer and colleagues have verified that the mechanism of cannabidiol against lung cancer may be related to the nuclear translocation of PPAR-*γ* by Cox-2-dependent prostaglandins (PG) and to the induction of PPAR-*γ*-dependent apoptosis cell death [47]. Using a combination of in vitro and computer simulation methods, Du L found that STAT3 and RAP1A can be utilized as direct targets to mediate the effect of miR-337-3p towards the sensitivity of paclitaxel to treat NSCLC [48]. Agarwal and colleagues have also found that the combination of the Jak1 inhibitor and silibinin (an active constituent of silymarin) is able to reduce STAT3 phosphorylation and to activate caspase-9 and caspase-3, thus leading to the apoptosis of prostate cancer DU145 cells [49]. The above findings are consistent with our experimental results where BEL can reduce the expression of CASP3, CASP8, PTGER2, EP4, COX-2, CEBPB, and STAT3. Therefore, BEL's MOA against lung cancer may involve a direct inhibition of STAT3/COX-2 signaling cascades, thereby affecting the caspase-dependent apoptosis pathway and, ultimately, cancer cell damage (Figure 19).

According to molecular docking analyses, we speculated that this anticancer effect driven by BEL could be related to its direct binding into the binding pocket of the STAT3/COX-2 receptor. Tyr705 phosphorylation of Stat3 leads to its dimerization and further nuclear translocation, which allows its binding to specific promoter sequences along a subset of JAK/STAT target genes [50]. Here, we found that BEL is capable of generating a static electricity with Ptr705 in the STAT3-SH2 domain binding region, thereby interfering with STAT3 dimerization. Rowlinson's studies have highlighted the importance of Tyr385 and Ser530 to the ligand association with COX-2 [51]. BEL can form hydrogen bonds with Tyr385 and Ser530 in the COX-2 receptor. This combination is equivalent to the recently reported effect of certain flavonoids towards COX-2 inhibition [52].

Taken together, we have presently found that BEL can efficiently inhibit the proliferation of lung cancer cells. According to our in vitro data, we speculate that BEL is capable of inducing apoptosis in human A549 lung cancer cells by upregulating the levels of caspase-8/3, reducing the level of PARP1, and ultimately, interfering into STAT3/COX-2 pathways. Furthermore, mechanistic studies focusing on the BEL's antilung cancer will strengthen the theoretical basis for the rational exploitation and utilization of the *Gentiana* phytoagents, thus broadening new views on the use of natural compounds as potential antitumor drugs. However, the therapeutic effect of BEL still requires further tests with other lung cancer cell lines to validate the current results and further in vivo studies.

## Figures and Tables

**Table 1:** qPCR primer sequences and product length of each respective amplicon.

Name of primers	Primer sequence (5′-3′)	Base number	Products length (bp)
GAPDH-F	ATGGGCAGCCGTTAGGAAA	19	118
GAPDH-R	GAGGAGAAATCGGGCCAGCTA	20	
STAT3-F	ACGACCTGCAGCAATACCAT	20	124
STAT3-R	AGGTGAGGGACTCAAACTGC	20	
COX-2-F	GTTCCACCCGCAGTACAGAA	20	106
COX-2-R	AGGGCTTCAGCATAAAGCGT	20	
PTGER2-F	AGGAGCTCCCTCTCCTTGTT	20	100
PTGER2-R	CGTACGAAGCCAGTACCACT	20	
EP4-F	GAGGCAGGAATTTGCTTCCAG	21	106
EP4-R	CCCTGTGAAGAGTCTGAGGTC	21	
C/EBPB-F	AACCTGGAGACGCAGCACAA	20	109
C/EBPB-R	TGAACAAGTTCCGCAGGGTG	20	

**Table 2:** Composition of qPCR reaction.

Component	Volume (*μ*L)
cDNA	3.0
Forward primer (10 *μ*m)	1.0
Reverse primer (10 *μ*m)	1.0
2x RealStar Green Power Mixture	5
Mix	10
TaqE	0.2
RNase-free H_2_O	4.8

**Table 3:** List of bellidifolin- (BEL-) related targets and respective topological parameter analysis.

Number	Name	Degree	Betweenness centrality
1	CDK5	2	0.200
2	CDK5R1	1	0
3	PTGS2 (COX-2)	6	0.222
4	STAT3	8	0.570
5	ESR1	6	0.059
6	TERT	4	0.015
7	PIM1	1	0
8	ESR2	3	0
9	RET	3	0
10	CA7	1	0
11	CA9	3	0

**Table 4:** Top diseases (*n* = 205) correlated with BEL-related targets.

Term	Count	*P* value	Gene IDs (NCBI)
Lung cancer	9	3.05*E* − 07	P03372, O14746, Q92731, P35354, P21397, P36544, P40763, P07949, and P11309
Premenstrual dysphoric disorder	3	3.04*E* − 05	P03372, Q92731, and P21397
Bladder cancer	7	7.70*E* − 05	P03372, O14746, Q92731, P35354, P21397, P07949, and P11309
Lung cancer	7	1.45*E* − 04	P03372, O14746, Q92731, P35354, P21397, P07949, and P11309
Breast cancer	7	1.52*E* − 04	P03372, O14746, Q92731, Q96GD4, P35354, P68400, and P40763

**Table 5:** Scoring of protein-protein binding interfaces.

Site name	Site scoring
Sitemap-14-site-3	1.258
Sitemap-14-site-2	1.120
Sitemap-14-site-4	1.096
Sitemap-14-site-1	1.014
Sitemap-14-site-5	0.859

Note: energy in Table 5 is in kilocalories/mole.

**Table 6:** Related indicators of BEL and STAT3 binding energy.

Ligand	Molecular formula	XP GScore	Glide emodel
BEL (bellidifolin)	C_13_H_8_O_6_ C_29_H_27_F_5_N_2_O_6_S	−3.271	−32.859
BP-1-102	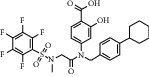	−3.606	−49.240

**Table 7:** Related indicators of bellidifolin and COX-2 binding energy.

Ligand	Molecular formula	XP GScore	Glide emodel
Bellidifolin	C_13_H_8_O_6_	−8.509	−41.973
Celecoxib	C_17_H_14_F_3_N_3_O_2_S 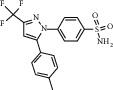	−11.288	−86.343

## Data Availability

The data used to support this study are included within this article.
